# Expression of Concern: Long non-coding RNA AK096174 promotes cell proliferation and invasion in gastric cancer by regulating WDR66 expression

**DOI:** 10.1042/BSR-20180277_EOC

**Published:** 2021-04-19

**Keywords:** AK096174, Gastric cancer, Invasion, Long noncoding RNA, WD repeat-containing protein

The Editorial Office has been made aware of potential issues surrounding the scientific validity of this paper, hence has issued an expression of concern to notify readers whilst the Editorial Office investigates.

